# Megacity pumping and preferential flow threaten groundwater quality

**DOI:** 10.1038/ncomms12833

**Published:** 2016-09-27

**Authors:** Mahfuzur R. Khan, Mohammad Koneshloo, Peter S. K. Knappett, Kazi M. Ahmed, Benjamin C. Bostick, Brian J. Mailloux, Rajib H. Mozumder, Anwar Zahid, Charles F. Harvey, Alexander van Geen, Holly A. Michael

**Affiliations:** 1 Department of Geological Sciences, University of Delaware, Newark, Delaware 19716, USA; 2 Geology and Geophysics, Texas A&M University, College Station, Texas 77843, USA; 3 Department of Geology, University of Dhaka, Dhaka 1000, Bangladesh; 4 Lamont-Doherty Earth Observatory, Columbia University, Palisades, New York 10964, USA; 5 Environmental Sciences, Barnard College, New York, New York 10027, USA; 6 Bangladesh Water Development Board, Government of Bangladesh, Green Road, Dhaka 1000, Bangladesh; 7 Department of Civil and Environmental Engineering, Massachusetts Institute of Technology, Cambridge Massachussetts, 02197, USA; 8 Department of Civil and Environmental Engineering, University of Delaware, Newark, Delaware 19716, USA

## Abstract

Many of the world's megacities depend on groundwater from geologically complex aquifers that are over-exploited and threatened by contamination. Here, using the example of Dhaka, Bangladesh, we illustrate how interactions between aquifer heterogeneity and groundwater exploitation jeopardize groundwater resources regionally. Groundwater pumping in Dhaka has caused large-scale drawdown that extends into outlying areas where arsenic-contaminated shallow groundwater is pervasive and has potential to migrate downward. We evaluate the vulnerability of deep, low-arsenic groundwater with groundwater models that incorporate geostatistical simulations of aquifer heterogeneity. Simulations show that preferential flow through stratigraphy typical of fluvio-deltaic aquifers could contaminate deep (>150 m) groundwater within a decade, nearly a century faster than predicted through homogeneous models calibrated to the same data. The most critical fast flowpaths cannot be predicted by simplified models or identified by standard measurements. Such complex vulnerability beyond city limits could become a limiting factor for megacity groundwater supplies in aquifers worldwide.

## Abstract (web summary)

Megacities rely on groundwater from aquifers that may be over-exploited and be at risk of contamination. Khan *et al*. evaluate the complex aquifers supplying Dhaka, Bangladesh and show that extensive groundwater pumping could lead to unpredictable future arsenic contamination in deep aquifers outside the city.

## Graphical abstract

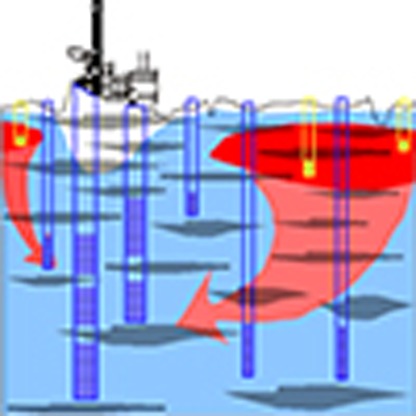

Deltas and river basins sustaining dense populations and unique ecosystems are sensitive to environmental stresses^1^. Nearly half a billion people, often concentrated in megacities, live in 50 deltas around the globe^2^. Groundwater is often used for water supply in deltas because of uncertainty in surface water availability and high vulnerability to contamination, as occurs in the Bengal Delta^3^. However, groundwater overdevelopment^4^,^5^,^6^, especially in megacities^7^,^8^,^9^, and groundwater contamination are common in deltas and river basins^10^,^11^,^12^,^13^. Under a changing climate, uncertainty of surface water supply is likely to increase^14^, and rapid population growth in urban centres^15^ may exacerbate groundwater depletion and contamination problems.

One consequence of pervasive groundwater over-development and depletion in city centres is an increase in vertical recharge in and around the city^11^,^12^,^16^,^17^ where the surface and near-surface waters are often contaminated with toxic metals, organics, nitrate and other pollutants^10^,^11^,^17^,^18^,^19^,^20^. Although sustaining the quantity and quality of city water supply is a high priority, little attention is given to the potentially catastrophic impacts these hydrologic alterations can have on the water resources of surrounding peri-urban and rural communities that do not benefit from city supply^21^, and for which water treatment may not be feasible.

Dhaka, the capital of Bangladesh, shares many of the water management problems common to major cities^22^ and is located in one of the largest fluvio-deltaic basins in the world. Naturally occurring arsenic in shallow groundwater (<50 m), the drinking water source for tens of millions of people, is widespread in the basin^18^, as in many fluvio-deltaic aquifers of Southern Asia^13^. Deep (>150 m), low-arsenic aquifers are increasingly relied on for reducing exposure of the rural population to arsenic and could be for decades to come if properly managed^23^,^24^,^25^. Dhaka pumping, however, has caused groundwater levels to drop more than 60 m over the last half century, and levels are currently declining at a rate of >3 m per year in areas of the city centre^7^,^26^. This massive alteration to the subsurface hydrology has expanded the management problem from local to regional because hydraulic heads are falling tens of kilometres beyond the city limits^16^. Over-pumping is lowering water levels beyond the threshold for handpump use^16^ and could induce downward migration of shallow groundwater^23^,^24^, which may transport arsenic and other contaminants or reactive organic carbon that can fuel reductive dissolution of iron (oxy)hydroxides and associated release of arsenic to groundwater^13^,^27^ from deeper, older sediments^28^. These risks threaten the sustainability of the deep groundwater resource of the 10 million people living in the Dhaka metropolitan area outside the city centre and in surrounding rural areas (Fig. 1) who are not supplied with city water.

Predicting the extent to which a hydrologic perturbation of this scale jeopardizes the quality of groundwater resources outside of pumping centres is confounded by geologic complexity. The large-scale heterogeneity characteristic of river basins and deltas creates groundwater flowpaths that can contaminate groundwater resources^29^,^30^, yet aquifers of this type host more than half of 47 indexed groundwater mega-depletion cases worldwide^6^. Typical of such systems, the aquifer system surrounding Dhaka is a highly heterogeneous, kilometres-thick sequence of fluvio-deltaic deposits centred in the 200,000 km^2^ Bengal Basin^31^. Numerous studies over the past four decades have addressed the influence of aquifer heterogeneity on transport of solutes, but many focus on small-scale, low-variance heterogeneity relevant to plumes observed on the scale of tens of metres^32^,^33^,^34^. Indeed, the importance of local heterogeneity in controlling transport of As and organic carbon has been demonstrated in As-affected aquifers of Cambodia^35^ and Bangladesh^36^,^37^,^38^. At the basin scale, however, we must consider heterogeneity in geologic features that spans tens of kilometres and many orders of magnitude in hydraulic conductivity (**K**)^39^. Predicting the vulnerability of groundwater on this large scale requires numerical modelling, but the complexity and unknown nature of the aquifer system often necessitates a simplified approach using spatially averaged aquifer properties^40^. This approximation may miss the small fraction of flowpaths which are fast and most relevant to water quality vulnerability^41^.

Here, we explicitly represent aquifer heterogeneity in a groundwater flow model to explore the existence and predictability of fast flowpaths, and associated vulnerability of groundwater resources to contamination around the Dhaka megacity pumping centre. Using lithologic data, we developed a statistical model that represents the lateral and vertical correlations among four lithofacies and simulated 60 equally probable realizations of aquifer heterogeneity on the 10 km scale, with smaller-scale structure represented as within-facies vertical anisotropy in **K**. We then simulated groundwater flow through these realizations and compared the results to a simplified, upscaled flow model in which an effective value for uniform, but anisotropic, **K** represented all scales of heterogeneity within the domain and reproduced the same bulk flow as the heterogeneous **K**-field. We show that concentrated groundwater pumping typical of megacities induces preferential flowpaths that threaten groundwater quality well outside the city limits.

## Results

### Effects on regional flow system

All of the simulations show that Dhaka pumping has fundamentally changed the natural hydrologic system, both within and far outside of the city centre (Fig. 2a,b). As a result, simulated net recharge increased to more than four times natural levels and rivers near the city changed from net gaining to net losing (Supplementary Figs 1 and 2). Both measured and modelled heads within the city centre are very low at all depths (Supplementary Figs 3–6). However, in surrounding areas up to 25 km from the city centre, deep (>150 m) hydraulic heads are more affected than those at shallow depths where groundwater is readily recharged from the surface, resulting in lower head at depth nearly everywhere in the study area (Fig. 2d).

### Implications for arsenic exposure

**Figure 1:**
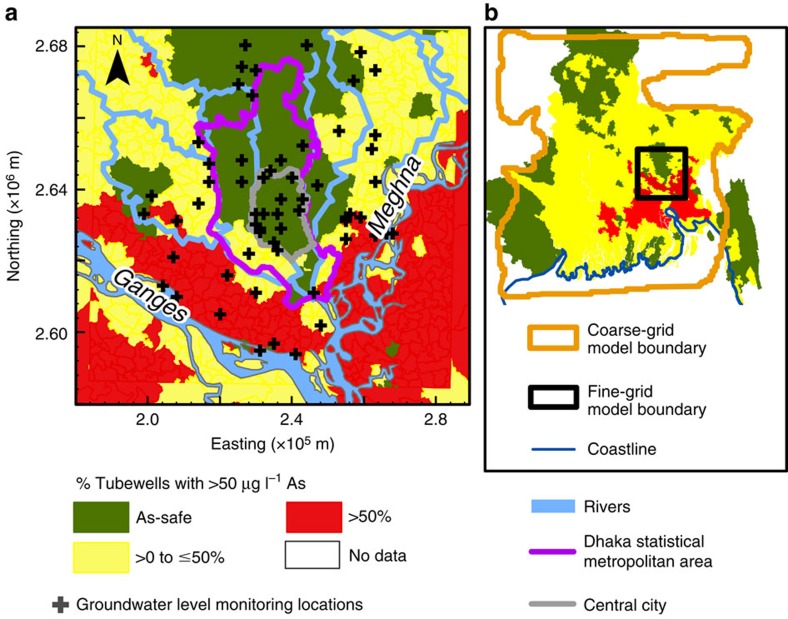
Map of the study area. Colours represent percentage of tubewells with >50 μg l^−1^ As (**a**) within the study area and (**b**) within the Bengal Basin. The orange polygon outlines the basin-scale (coarse-grid) model and the black rectangle outlines the locally refined (fine-grid) model. Arsenic data sources: DPHE-JICA^59^ for Bangladesh and Chakraborti *et al*.^60^ for West Bengal. DWASA only supplies municipal water within the central city area.

**Figure 2:**
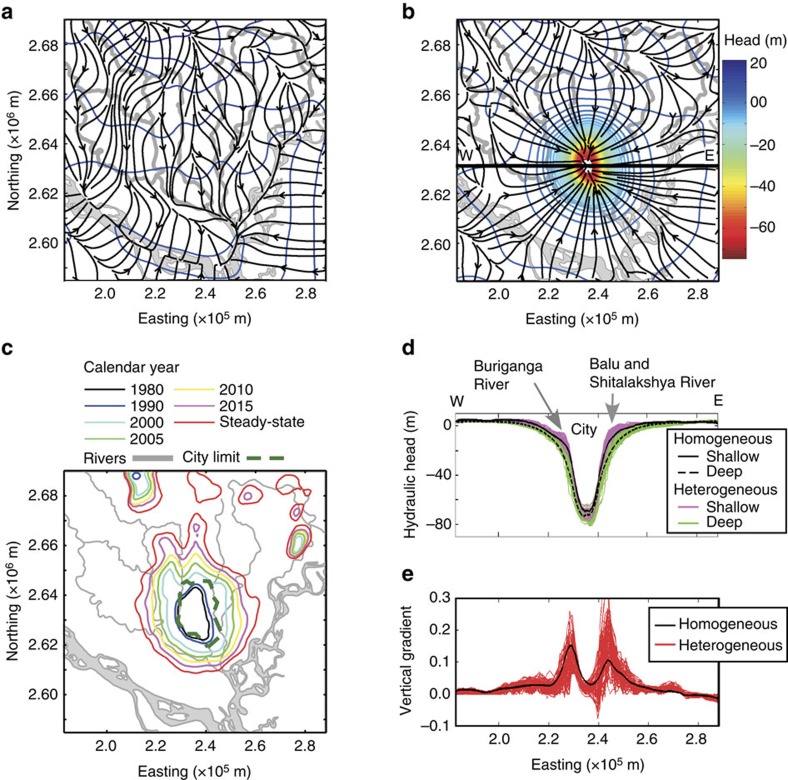
Effects of Dhaka pumping on the flow system. Hydraulic head (contours) and flowpaths (black arrows) within the study area simulated for homogeneous conditions at 150 m depth for (**a**) pre-development (before 1980) and (**b**) current pumping (2015) conditions. (**c**) Extent of 9 m water level depth (suction limit for handpump wells) at 150 m depth. The steady-state condition in **c** is based on current Dhaka pumping levels. (**d**,**e**) Cross-sectional views of hydraulic head at depths of 50 m (shallow) and 150 m (deep) and the vertical gradient, respectively. Cross-sections are shown along the W–E transect shown in **b**.

**Figure 3:**
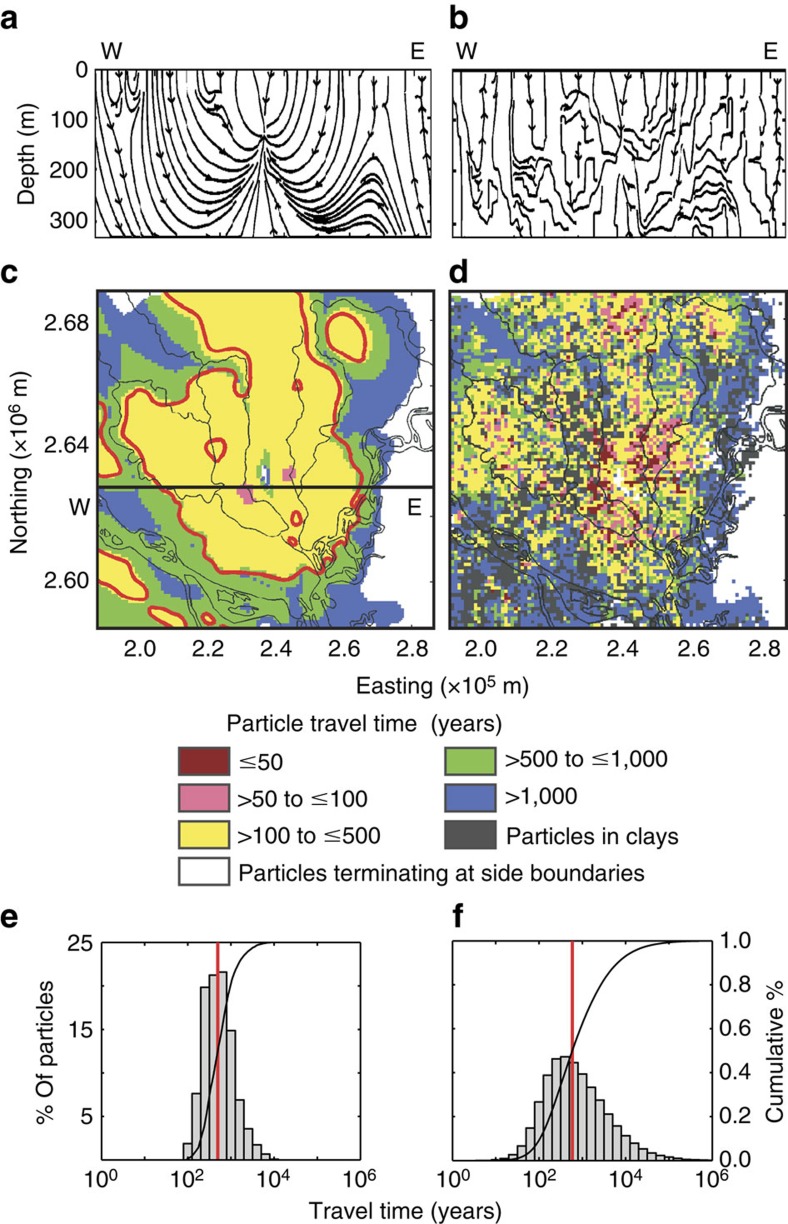
Comparison of flowpaths and travel times for homogeneous and heterogeneous aquifer systems. Cross-sectional views along the W–E line in panel **c** of streamlines for the (**a**) homogeneous and (**b**) heterogeneous model simulations. Simulated travel time for (**c**) homogeneous model and (**d**) a heterogeneous model (Realization 1). Travel times are determined by backtracking particles from 150 m depth at every model cell to 50 m depth. White indicates areas where particles terminate on the lateral boundaries of the fine-grid model (distant recharge). The red line in **c** is the contour within which vertical hydraulic gradient is >1.3 × 10^−2^. Histograms and cumulative distribution functions of simulated particle travel times for (**e**) the homogeneous case and (**f**) all 60 heterogeneous realizations. In **e** and **f**, the red line indicates the median travel time. Only particles initially located in sands and terminating within the fine-grid model are included in **e** and **f**.

There are two important consequences of this altered groundwater system. First, deep handpump tubewells, installed to mitigate exposure to high levels of arsenic in shallow groundwater in outlying areas without access to city water supply, are becoming inoperable because the lift required to bring water to the surface is greater than can be provided by suction handpumps (∼9 m; ref. 16). Our simulations indicate that should pumping continue at current levels, hydraulic heads would equilibrate such that handpumps on deep wells would be inoperable within a ∼25 km radius from the city centre within 10 years, affecting an area of 2,400 km^2^ (Fig. 2c) and a population of over 5 million outside the city centre. Second, the induced vertical hydraulic gradient (Fig. 2e) is driving downward flow in much of the study area (Fig. 3a,b). Backward particle tracking shows that in many areas outside the city, low-arsenic groundwater at 150 m depth originates in shallow zones (Fig. 3c,d) where arsenic concentrations are high (Fig. 1).

### Aquifer heterogeneity and deep aquifer sustainability

The homogeneous and heterogeneous models yield essentially the same groundwater flow directions (Fig. 3a,b), recharge amounts (Supplementary Fig. 2) and locations, and simulated hydraulic heads (Fig. 2d) across the 61 simulations, but the similarities are deceptive. Local vertical gradients are highly variable in heterogeneous cases (Fig. 2e), as are the distributions of travel time between 50 and 150 m depth (Fig. 3c–f, Supplementary Figs 7 and 8). Although the median travel times are similar for both homogeneous and heterogeneous aquifer representations (Table 1), the tails of the distributions are much different (Fig. 3e,f, Supplementary Figs 7 and 8). Importantly, there are some flowpaths through heterogeneous fields that are very short (Supplementary Fig. 9). The minimum travel time predicted by the simplified homogeneous model is 89 years. In contrast, in the heterogeneous simulations, 8% of travel times are shorter than 89 years, with a minimum of only 7 years. Travel times <100 years exist in an average of 9% of locations in heterogeneous simulations, compared with <0.5% in the homogeneous case (Fig. 3c,d and Table 1). In these heterogeneous aquifers, nearly all of the flow in the lateral direction and >95% of the flow in the vertical direction occurs through medium to coarse sands (Supplementary Fig. 10). Thus, the connectivity of high-**K** sediments determines the length of the flowpaths, a small proportion of which are short connections between contaminated and uncontaminated depths.

**Figure 4:**
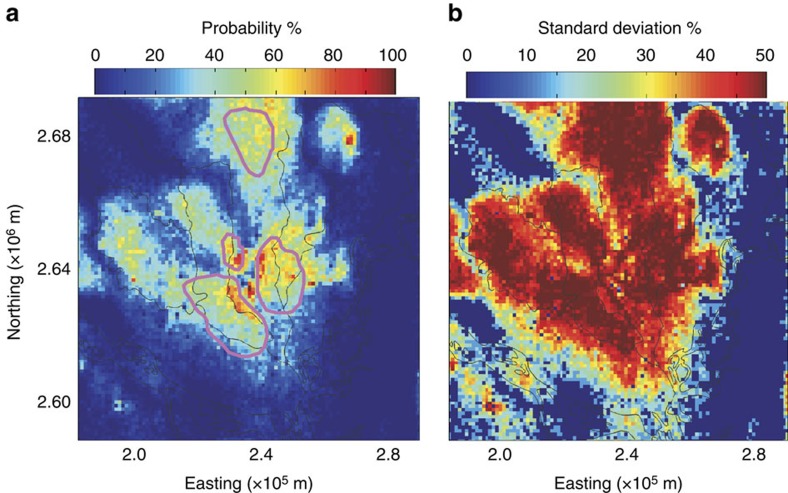
Vulnerability uncertainty. Spatial distribution of (**a**) probability and (**b**) standard deviation of the deep (≥150 m) aquifer becoming contaminated in ≤200 years based on the travel times of 60 heterogeneous realizations. Contours in **a** show the area in the homogeneous simulation within which particle travel time is ≤200 years. Standard deviation of this binary variable (safe or unsafe in 200 years) is 50% where it is equally likely to be safe or unsafe, and ≤30% where likelihood of being safe is ≥90% or ≤10%.

The complexity of flow through fluvio-deltaic sediments creates high variability such that for the same hydrologic forcing, vulnerability varies widely. The probability of contamination at 150 m depth within 200 years was determined over the 60 equally probable heterogeneous simulations (Fig. 4a). If we consider probability of contamination to be highly likely or highly unlikely if >90% of realizations have flowpaths at a location that are unsafe or safe, respectively, then only 0.05% of the area is highly likely to be contaminated, and 56% of the area is likely safe. In the rest of the study area, represented by a standard deviation of >30% in Fig. 4b, uncertainty is high.

### Unpredictable contamination pathways

**Figure 5:**
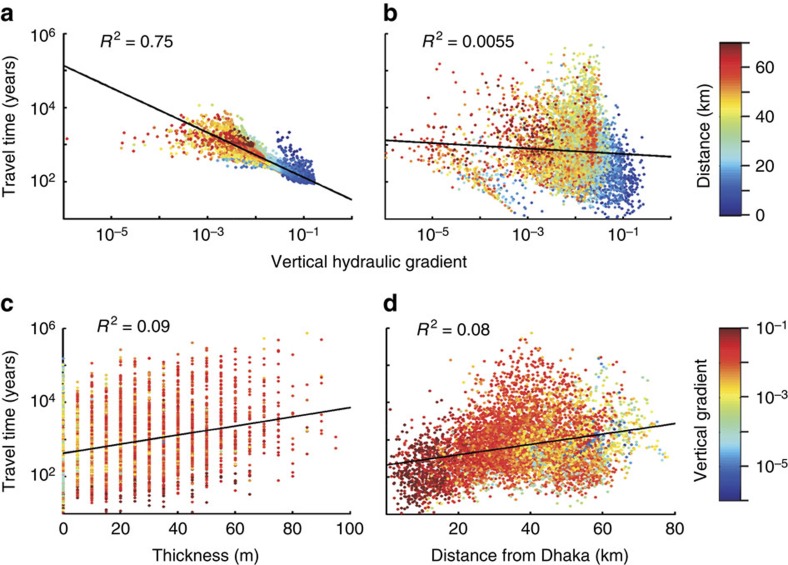
Correlation between travel time and vertical gradient between 50 and 150 m depth for the simplified homogeneous system. Particle travel time versus vertical hydraulic gradient for (**a**) homogeneous model and (**b**) heterogeneous realization. In **a** and **b**, colour represents distance of the particle location to Dhaka city centre. Correlation of particle travel time with (**c**) thickness of overlying fine sediments and (**d**) distance from Dhaka. The black line in each panel is a linear regression.

An added complication that results from heterogeneous stratigraphy is that no local hydrogeologic features serve as a reliable indicator of vulnerability. In the simplified homogeneous model, the local vertical hydraulic gradient is a strong indicator of vulnerability (Figs 3c and 5a). However, aquifers are heterogeneous and, with the realistic representations of heterogeneity used for our simulations, particle travel time does not correlate with either the vertical hydraulic gradient or total thickness of fine sediments in the vertical stratigraphic section between 50 and 150 m depth, as can be observed in cores or driller logs (Fig. 5b,c). This finding is consistent with local measurements that display a similar lack of correlation between groundwater age (based on ^14^C and ^3^H) and the thickness of overlying clay^42^. Exceptions are locations with no fine sediments in the stratigraphic column. There, the vertical gradients are too small (<0.001) to be measured reliably using local methods, yet many travel times are short (Supplementary Fig. 11). These areas should always be considered vulnerable unless a gradient driving upward flow is evident. Proximity to the pumping centre, athough an important factor, also does not reliably predict travel time (Figs 3d and 5d). This unpredictability is likely because the combination of concentrated pumping and the spatial distribution of fine and coarse sediments creates tortuous, three-dimensional preferential flow paths (Fig. 3b, Supplementary Fig. 9).

## Discussion

This unpredictability of contaminant migration in heterogeneous aquifers is likely to be exacerbated by other factors. The modelling analysis includes only advective transport and heterogeneity at scales larger than the grid blocks. Physical heterogeneity beneath the scale represented in our models may result in earlier arrival times of solute through additional dispersive processes. Sorption has been shown to slow arsenic transport^25^, but heterogeneity in the sorptive properties of aquifer sediments has not been well characterized. Thus, our simplified approach may underpredict uncertainty in vulnerability.

Although uncertainty may be underestimated, this analysis likely overpredicts the vulnerability to arsenic migration because sorption and other reactions that may immobilize arsenic and retard its movement are neglected. Laboratory-based estimates of the sorptive properties of Bengal Basin sediments vary greatly^43^,^44^,^45^,^46^, but *in situ* estimates of arsenic retardation factors in the deep Pleistocene sediments of the Bengal Basin^25^ are 13 to up to 110, and 16–20 in a similar Pleistocene aquifer in Vietnam^28^. This means that in the absence of preferential low-sorption flow pathways, breakthrough will likely be greatly delayed in deeper Pleistocene parts of the flow system relative to the advective travel times considered here. More extensive characterization of sediment chemistry will improve our ability to predict the evolution of As concentrations in vulnerable areas. However, preferential transport of more conservative contaminants or reactive organic carbon in this and other systems may be less delayed by reaction.

In many highly populated deltas and river basins worldwide, water resources are stressed and surface pollution is widespread. We show that pumping to supply water to megacities in these regions, even in the water-rich system of the Bengal Basin, threatens the safety of regional groundwater resources by inducing fast, preferential transport of contaminants to depth, even in areas tens of kilometres outside the city limits. We demonstrate that traditional indicators of vulnerability to contamination may not be predictive in thick fluvio-deltaic aquifers and that pumping may impact groundwater quality more quickly than anticipated. We also establish that simplified models can replicate hydraulic head distributions and physical hydrologic changes while failing to fully predict vulnerability to solute transport in the presence of intensive pumping. These uncertainties are particularly worrying when detrimental effects extend far beyond the area where populations receive city resources.

These findings have important implications for both hydrogeologic analysis of contaminant migration and for city water management. First, even the best-calibrated models can miss the most relevant information if the potential for preferential flow is neglected. Though this is a well-known problem in contaminant transport, this example illustrates that it is amplified in the presence of intensive pumping in a three-dimensional flow field. Second, municipal water managers must consider not only the challenge of providing enough water to a dense population, but also the impacts of its extraction on both the quantity and quality of water in the region beyond the city. Because hydrogeologic data cannot be used to predict the locations of preferential flow, conservative management would avoid all vulnerability. However, this is usually not possible in practice, and prevention of health effects will require extensive water quality monitoring programmes, potentially operating outside of city management districts. Unfortunately, detection will indicate that aquifers are already compromised.

## Methods

### Flow modelling

A groundwater flow model was developed from an existing MODFLOW^47^ model of the Bengal Basin^24^,^48^,^49^ that was refined locally in the study area (Supplementary Fig. 12) with the MODFLOW local grid refinement (LGR2) package^50^. The basin-scale model encompasses the permeable sediments of the Bengal Basin. Boundary conditions outside the study area were specified head at the land surface, representative of a water table near the surface^23^,^48^,^49^. Within the study area, pumping has lowered the water table; therefore, the surface boundary condition was specified recharge with constant head boundaries along the rivers within the embedded fine-grid model. In this area, a constant recharge rate of 0.5 m per year was used with a drain boundary that removes excess recharge that would result in a water table above land surface. The simulated recharge is within the range of hydrograph-based regional recharge estimates^3^ and isotope-based local recharge estimates^51^,^52^ in the study area. All other model boundaries were zero flux^24^,^48^,^49^, corresponding to either the impermeable hard rocks in the north, west and east, or the offshore subsea aquifer in the south (Supplementary Fig. 12). The model bottom was also a zero flux boundary corresponding, where its location could be mapped, to the very low permeability Upper Marine Shale member of the Bokabil Formation^31^. The boundary fluxes along the sides and bottom of the fine-grid model were calculated iteratively by the MODFLOW-LGR2 package^50^.

The coarse-grid, basin-scale model consisted of 124 rows, 117 columns and 37 layers. Each of the columns and rows were of equal width, 5 km, and the layers varied in thickness. The embedded fine-grid model was 105 km × 105 km × 326 m and centred on Dhaka city (Supplementary Fig. 12). It consisted of 105 rows and 105 columns of equal width, and 66 layers, each 5 m thick except the top layer which was 1 m thick.

Outside the study area, hydraulic conductivity (**K**) was represented as homogeneous, with horizontal and vertical values representative of the large-scale system, **K**_h_=5 × 10^−4^ m s^−1^ and **K**_v_=5 × 10^−8^ m s^−1^, a result of basin-scale model calibration^48^,^49^. This anisotropy incorporates effects of heterogeneity that are not explicitly represented. Inside the study area, both homogeneous and heterogeneous representations were used. The homogeneous **K**_h_ and **K**_v_ values within the study area were calibrated and validated against historic records (median record length of 21 years with a range from 3 years to 28 years between 1986 and 2015) of weekly measured hydraulic head data at 75 monitoring stations at variable depths (7–277 m) located within the fine-grid model (see ‘Calibration' section below; Supplementary Fig. 3). The calibrated values of **K**_h_ and **K**_v_ for the homogenous system were 2.0 × 10^−4^ and 1.0 × 10^−7^ m s^−1^, respectively. The storage coefficient was 1.0 × 10^−4^, with the exception of the top layer, where it was 0.1, corresponding to a value of specific yield divided by cell height (1 m). This mimics unconfined conditions despite using MODFLOW confined conditions to avoid cell drying. Comparison with simulations with unconfined conditions that allowed cell drying indicated the maximum error associated with that assumption to be 8 m (10% of head). However, errors were negligible in the area of interest beyond the city boundaries (Supplementary Fig. 6), so confined conditions were retained.

The simulations were transient with 38 stress periods. The first and last stress periods were steady-state, the remaining 36 annual stress periods represented the time period from 1980 to 2015. For each stress period, the total annual groundwater pumping was assigned in terms of area using the MODFLOW Well Package. A steady-state flow simulation for the period prior to 1980, when pumping began, was the initial condition for transient simulation from 1980 to 2015. In heterogeneous models, the aquifer zones within the pumping depth intervals within the study area were assigned homogenous equivalent values to avoid unrealistic drawdown due to pumping within clays.

Domestic and irrigation pumping rates were estimated on the basis of either upazila-wise (for the greater Dhaka metropolitan area) or district-wise (for the rest of the basin) data of population density and irrigated area, respectively, following the procedure in Michael and Voss^24^,^48^,^49^. Outside the city, pumping was assigned between 15 and 50 m depth, the range of most pumping in rural areas. Within Dhaka city, pumping was assigned on the basis of data from the Dhaka Water Supply and Sanitation Authority^26^ between 50 and 150 m depth before 2004. From 2004 onward, some of the wells were deepened due to large drawdowns, so a portion of the total pumping was assigned between 170 and 270 m depth. Population data are available for census years 1981, 1991, 2001 and 2011 (Bangladesh Bureau of Statistics Population Census) and the irrigation area data are available for irrigation census year 1996, and 2008 (Bangladesh Bureau of Statistics Agriculture Census). Population between census years was determined using the calculated population growth rate between census years. A linear growth rate was used to calculate the irrigated area between census years 1996 and 2008. For the period before 1996, the 1996 irrigated area data were used, and for the period after 2008, the 2008 irrigation area data were used.

### Model calibration and validation

The homogeneous, anisotropic model was calibrated against historic records (record length varies from 19 to 28 years between 1986 and 2014) of weekly measured hydraulic head data at eight monitoring stations of the Bangladesh Water Development Board at depths between 75 and 100 m located within Dhaka city (Supplementary Figs 3 and 4). The simulated head at each location was compared with the mean annual head for that station (Supplementary Fig. 4). In addition, the model was calibrated against continuous head measurements made for this study and Knappett *et al*.^16^, using data loggers at depths from 150 to 280 m along the transect shown in Supplementary Fig. 3 for the year 2014 (Supplementary Fig. 5b). This allowed us to constrain the shape of the cone of the depression in the deeper part of the aquifer system. We also used projected head from observations made by IWM & DWASA^26^ at three locations on the transect (circled in Supplementary Fig. 5b).

The calibrated model was then validated against hydraulic head measurements made in this work and obtained from the Bangladesh Water Development Board (record length varies from 3 years to 28 years between 1986 and 2014) at 64 locations within the fine-grid model (Supplementary Fig. 3). There was good agreement between the observed and simulated heads for both the homogeneous (Supplementary Fig. 5a) and the heterogeneous cases (Supplementary Fig. 5c). The overall root mean square error for the observed versus simulated head is about 3.5 m, which is within the seasonal variation in the observed data.

### Geostatistical simulations of aquifer heterogeneity

Sixty realizations of heterogeneous aquifer stratigraphy (Supplementary Fig. 13) were generated with sequential indicator simulation^53^. Variograms were developed from and simulations conditioned to lithological data from 433 driller logs obtained in this work and from IWM & DWASA^26^,^54^, DPHE and JICA^55^, MacDonald^56^ (Supplementary Figs 12 and 13). The lithologies from driller log descriptions were categorized into four lithofacies: clay, silt, very fine and fine sand, and medium and coarse sand, with proportions 13, 15, 26 and 46%, respectively.

Variogram models were fit to the experimental variograms derived from the lithologic data. The variogram model used for each lithofacies is:


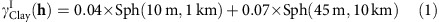



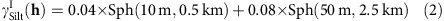



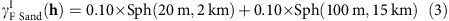



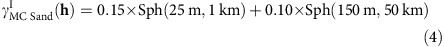


In equations 1, 2, 3, 4, the first number in parentheses is the range of the variogram in the vertical direction and the second is the range in the horizontal direction; Sph is a standard spherical variogram model. The search ellipse used in simulation had a horizontal radius of 10 km and a vertical radius of 30 m. An example simulated field is shown in Supplementary Fig. 13e.

Each of the simulated lithofacies was assigned a **K**_h_ and a **K**_v_ value typical of that sediment type. Using a MATLAB script, these facies **K** values were adjusted until the equivalent **K**_h_ and **K**_v_ of each of the heterogeneous cases, as determined by numerical simulation of Darcy flow horizontally and vertically across the domain, were within 20% of that of the homogeneous, calibrated values. These are summarized in Supplementary Table 1 and Supplementary Fig. 14. Most of the boreholes in the study area were drilled using a reverse circulation rotary method, commonly known as the donkey method. The sediment samples collected at the terminal of the fluid circulation system usually contain a mixture of sediments over the sampling depth intervals (∼3 m). This tends to under-sample thin lenses of silt and clay, which are easily washed away. Chances of missing these thin lenses of fine sediments are greater for intervals of very fine and fine sands, because the likelihood of coexisting silt and clay is greater with fine sands than for medium to coarse sands. These thin lenses of small lateral extent would likely affect the vertical flow more than the horizontal flow. Therefore, vertical anisotropy (**K**_h_/**K**_v_) values of 10 and 100 were applied for medium and coarse sands and very fine and fine sands, respectively. Hydraulic heads simulated by heterogeneous and homogeneous models are similar (Fig. 2d and Supplementary Fig. 5); heads are less sensitive to local heterogeneity than to effective aquifer properties.

### Simulation of advective transport

MODPATH^57^ particle tracking was used to determine the travel time and flowpaths of water reaching a depth of 150m, approximately the shallowest depth of deep wells drilled for arsenic mitigation^58^. The particles were tracked backwards from 150 m depth to either 50 m depth, the approximate lower boundary of the highly arsenic-contaminated zone^18^, or at the lateral and bottom boundaries of the fine-grid model. Travel times of particles that terminated at the lateral and bottom boundaries of the locally refined grid were not included in the analysis, as these likely have very long travel times and terminate in low-arsenic areas. We note that while we focus on 150 m depth because it is considered widely As-safe, many wells draw water from low-As zones in intermediate (50–150 m) depths, and travel times to these depths would be less.

Many particles were originally located in silts and clays, which led to significant tailing effects (Supplementary Fig. 7). Since groundwater is pumped primarily through high-permeability sands, we also plotted the distribution of travel times using only particles originating in medium to coarse sands. This resulted in less significant tailing (Supplementary Fig. 7), though it was still greater than the homogeneous case. All of the subsequent results are reported only for particles initially located in sand. The summary statistics of particle travel time are shown in Supplementary Table 2 and Supplementary Fig. 8.

### Sensitivity analysis

The horizontal range for the lithofacies variogram is the parameter with the largest uncertainty in our geostatistical model. To consider how this uncertainty may affect the results, we performed a sensitivity analysis to quantify the impact of the horizontal variogram range on simulated hydraulic heads and advective travel time distributions. Two additional sets of heterogeneous realizations (60 in each set) were simulated, one with a horizontal variogram range half of the original case and one with twice the range. Variograms of all of realizations of the three sets are shown in Supplementary Fig. 15. Facies **K**_h_ and **K**_v_ values were varied such that all realizations have the same equivalent **K**_h_ and **K**_v_ as the calibrated homogeneous model, regardless of the variogram range. All of the simulated fields were conditioned to the 433 lithologic logs. Because of the high density of points used to condition the realizations, the facies **K**_h_ and **K**_v_ values were similar across realizations (Supplementary Table 1). Furthermore, the horizontal variogram range had a small effect on both the simulated heads and travel times (Supplementary Figs 16 and 17, and Supplementary Table 2). Thus, the sensitivity analysis provides some confidence that the geostatistical simulations are sufficiently constrained by field data.

### Data availability

The numerical models and data original to this study are available from the authors.

## Additional information

**How to cite this article:** Khan, M.R. *et al*. Megacity pumping and preferential flow threaten groundwater quality. *Nat. Commun.* 7:12833 doi: 10.1038/ncomms12833 (2016).

## Supplementary Material

Supplementary Information Supplementary Figures 1-17, Supplementary Tables 1-2

## Figures and Tables

**Table 1:** Summary particle travel time statistics from 50 to 150 m depth.

**Particle statistics**	**Homogeneous**	**Ensemble mean (heterogeneous realizations)**
Minimum	89	7
Mean (fastest 10%)	131	67
1st quartile	291	220
Median	496	589
Mean	747	6,736
3rd quartile	846	1,959
Maximum	1.2 × 10^4^	1.5 × 10^7^
% Travel time <100 years	0.5	9.2

The statistics are based on only those particles that were originally placed in sands and ended inside the fine-grid model. Travel times are in years.
