# Supplementary material for: Megacity pumping and preferential flow threaten groundwater quality
Source: Nat Commun. 2016 Sep 27;7:12833. doi: 10.1038/ncomms12833 (PMC5052627; doi:10.1038/ncomms12833)
Supplement: Supplementary Information — Supplementary Figures 1-17, Supplementary Tables 1-2 [file ncomms12833-s1.pdf]

SUPPLEMENTARY INFORMATION

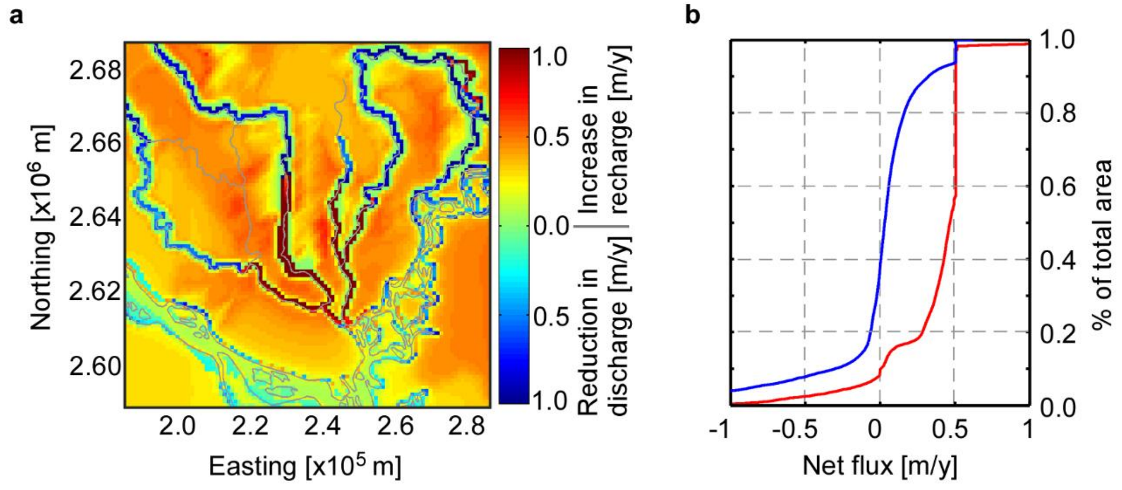

Supplementary Figure 1| Effects of Dhaka pumping on vertical groundwater recharge. (**a**) Spatial distribution and (**b**) cumulative distribution functions of the changes in simulated vertical recharge and groundwater discharge across the model surface between pre-development (before 1980) and current (2015) conditions.

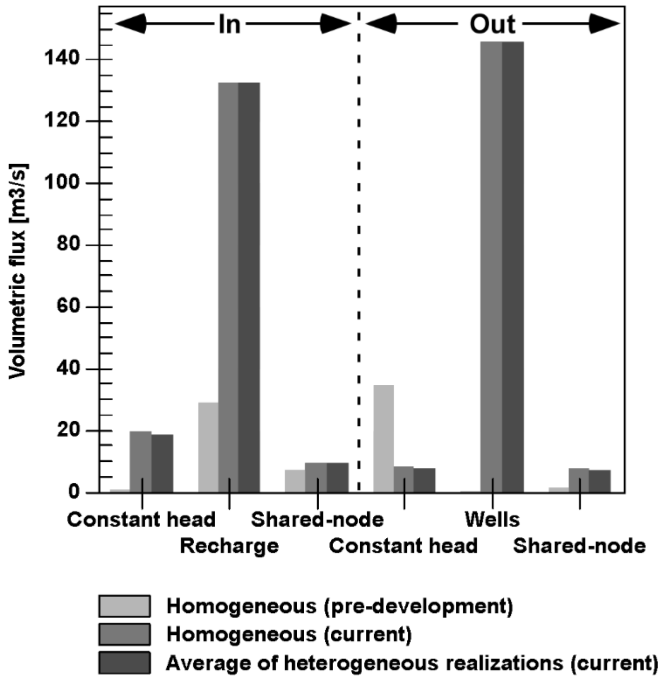

Supplementary Figure 2| Water budget for the entire model area at steady-state with current pumping rate. Shared-nodes are the common nodes between the fine-grid model and the coarse-grid regional model.

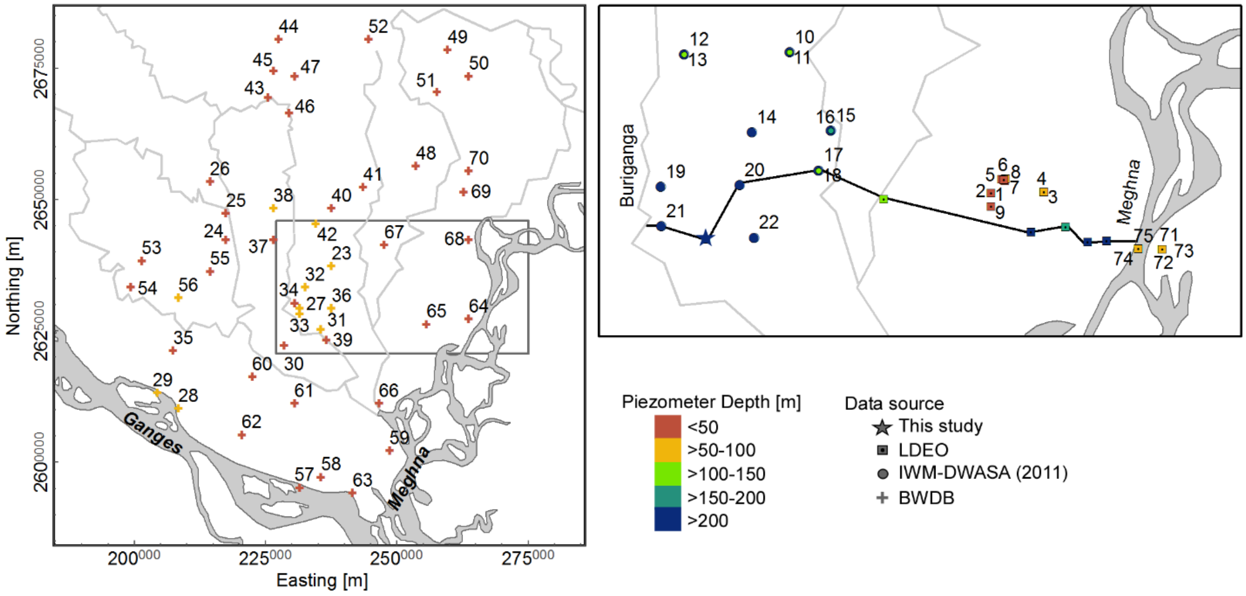

Supplementary Figure 3| Piezometer location map. Location of hydraulic head measurements used for model calibration and validation.

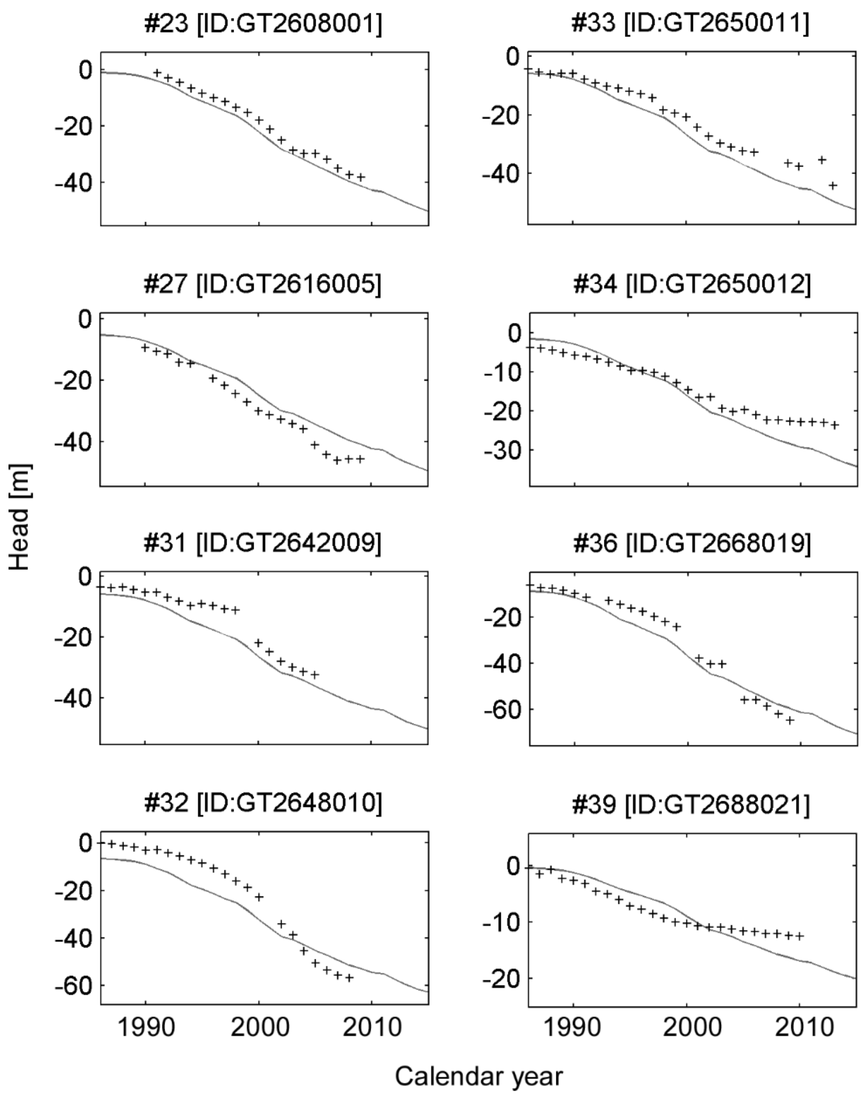

Supplementary Figure 4| Example model calibration plots. Data locations are shown in Supplementary Fig. 3.

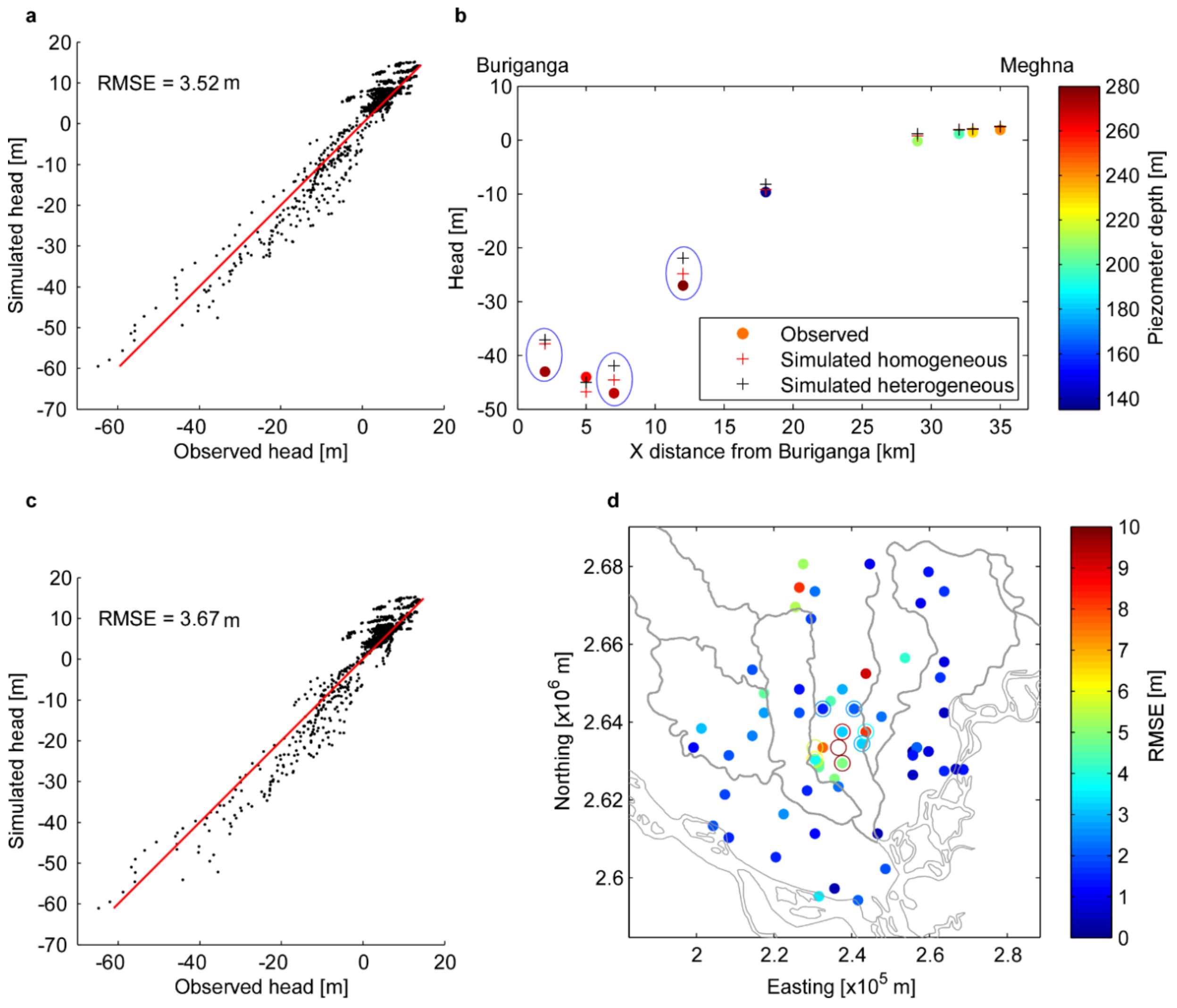

Supplementary Figure 5| Model calibration results. (**a**) Simulated (homogeneous model) vs. observed hydraulic head, (**b**) Simulated deep hydraulic head in homogeneous model vs. observed deep hydraulic head along the transect (shown in Supplementary Fig. 3), (**c**) Simulated (heterogeneous realization 1) vs. observed hydraulic head. Panel (**c**) is representative of all the heterogeneous realizations as the simulated hydraulic heads do not vary much among the different heterogeneous realizations. (**d**) Root mean square error at each measurement location for the homogeneous case.

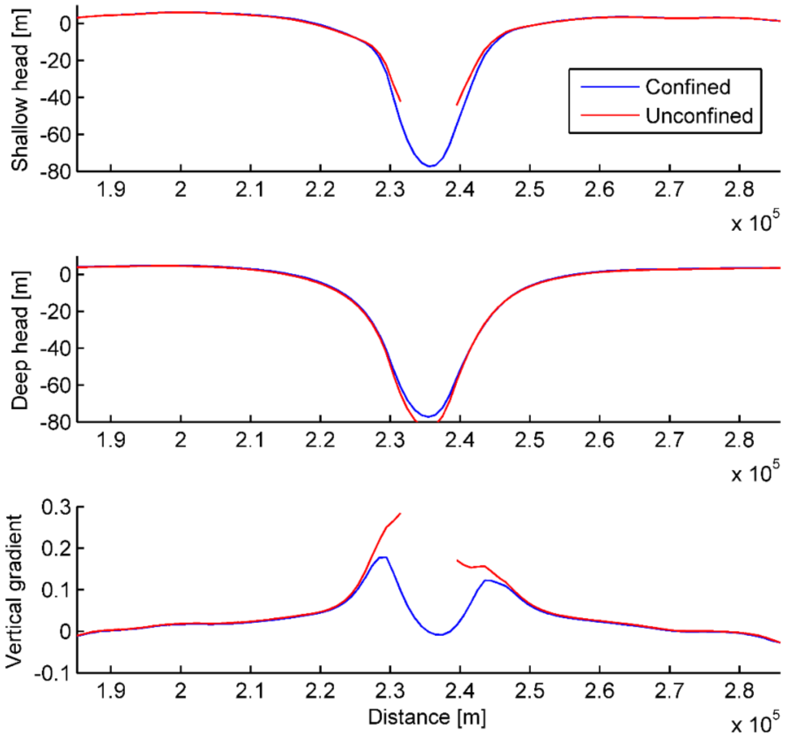

Supplementary Figure 6| Comparison of unconfined and confined simulation. Simulated hydraulic head (shallow at 50 m depth, deep at 150 m depth) and average vertical hydraulic gradient along a W-E transect through the center of Dhaka city (Fig. 2b) for the steady-state confined and MODFLOW unconfined simulations. The abrupt termination of red lines indicates dry cells in shallow aquifer depths.

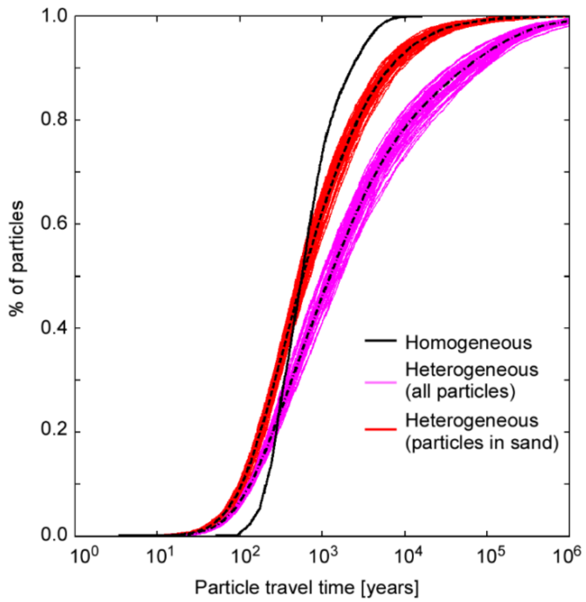

Supplementary Figure 7| Breakthrough curves of particle travel times. Travel time to 150 m depth from 50 m depth based on backward particle tracking. For the heterogeneous cases multiple lines represent the sixty realizations. The dashed line and the dashed and dotted line are cumulative distribution functions of the ensemble of the 60 realizations for particles originating in sand and all particles, respectively.

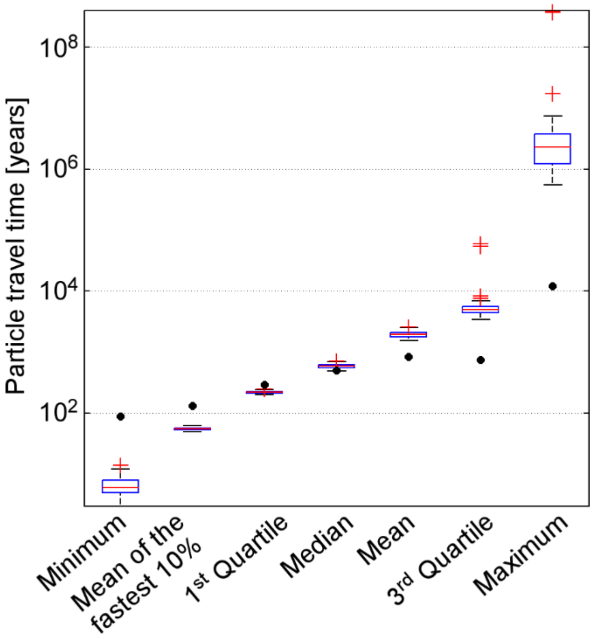

Supplementary Figure 8| Comparison of particle travel time statistics for homogeneous and heterogeneous realizations. The plots are based on only those particles that were initially located in sand and ended inside the fine-grid model for the homogeneous and each of the sixty heterogeneous realizations. Box plots show median (red line), range (whiskers), and outliers (red +) for heterogeneous realizations and black dots represent the homogeneous simulation.

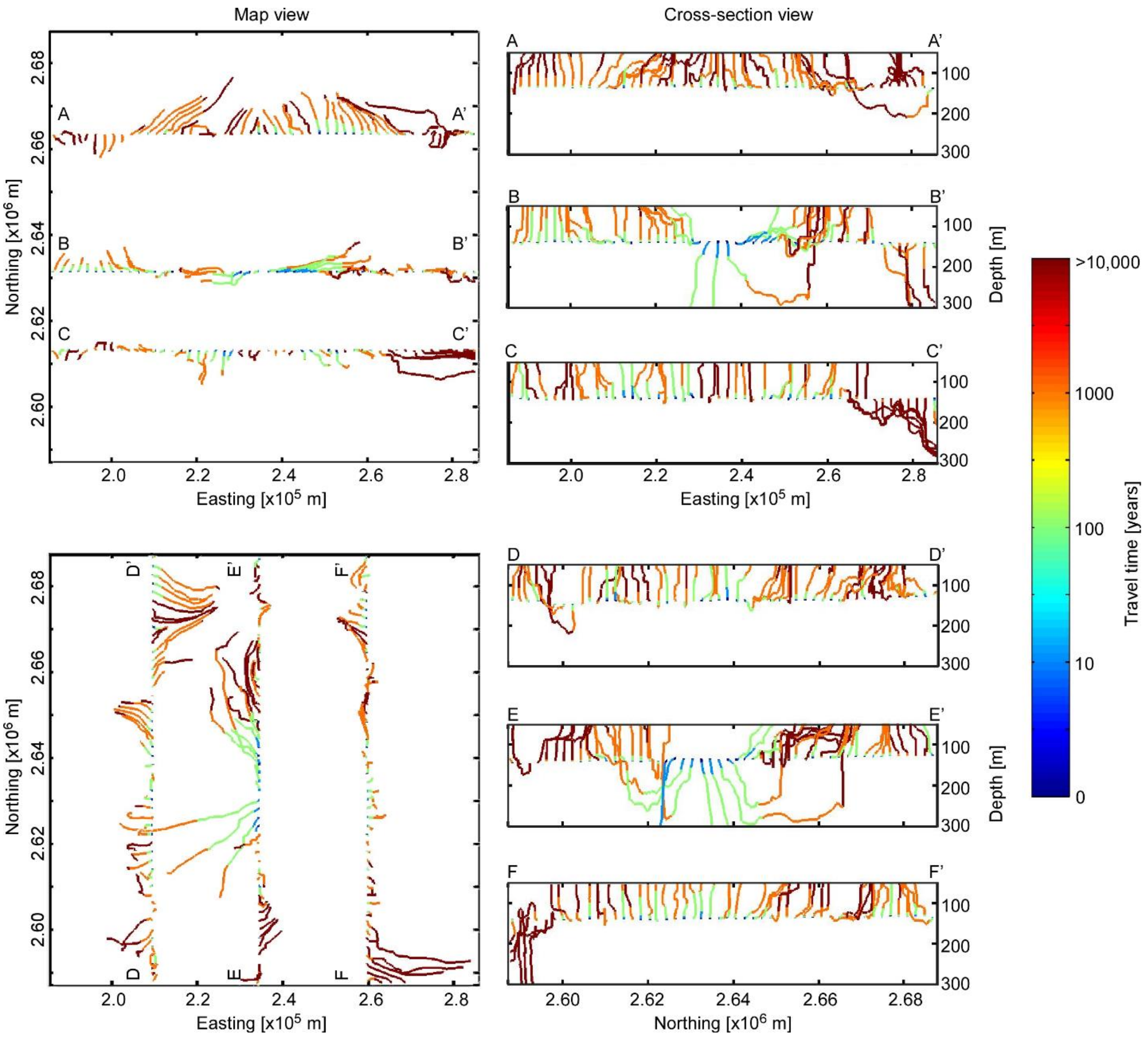

Supplementary Figure 9| Particle flowpaths along selected transects. The plots show flowpaths for heterogeneous Realization 1.

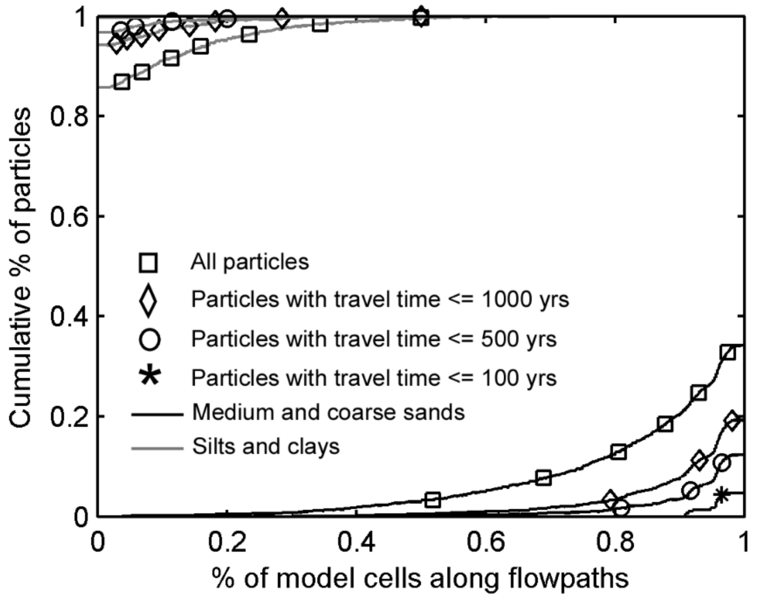

Supplementary Figure 10| Evaluation of preferential flowpaths. Percentage of model cells containing medium and coarse sands (black lines) and silts and clays (gray lines) along the particle flowpaths for different travel time categories. For example, along flowpaths with travel times <=100 years, more than 90% of the model cells are medium and coarse sands, and none are silts and clays.

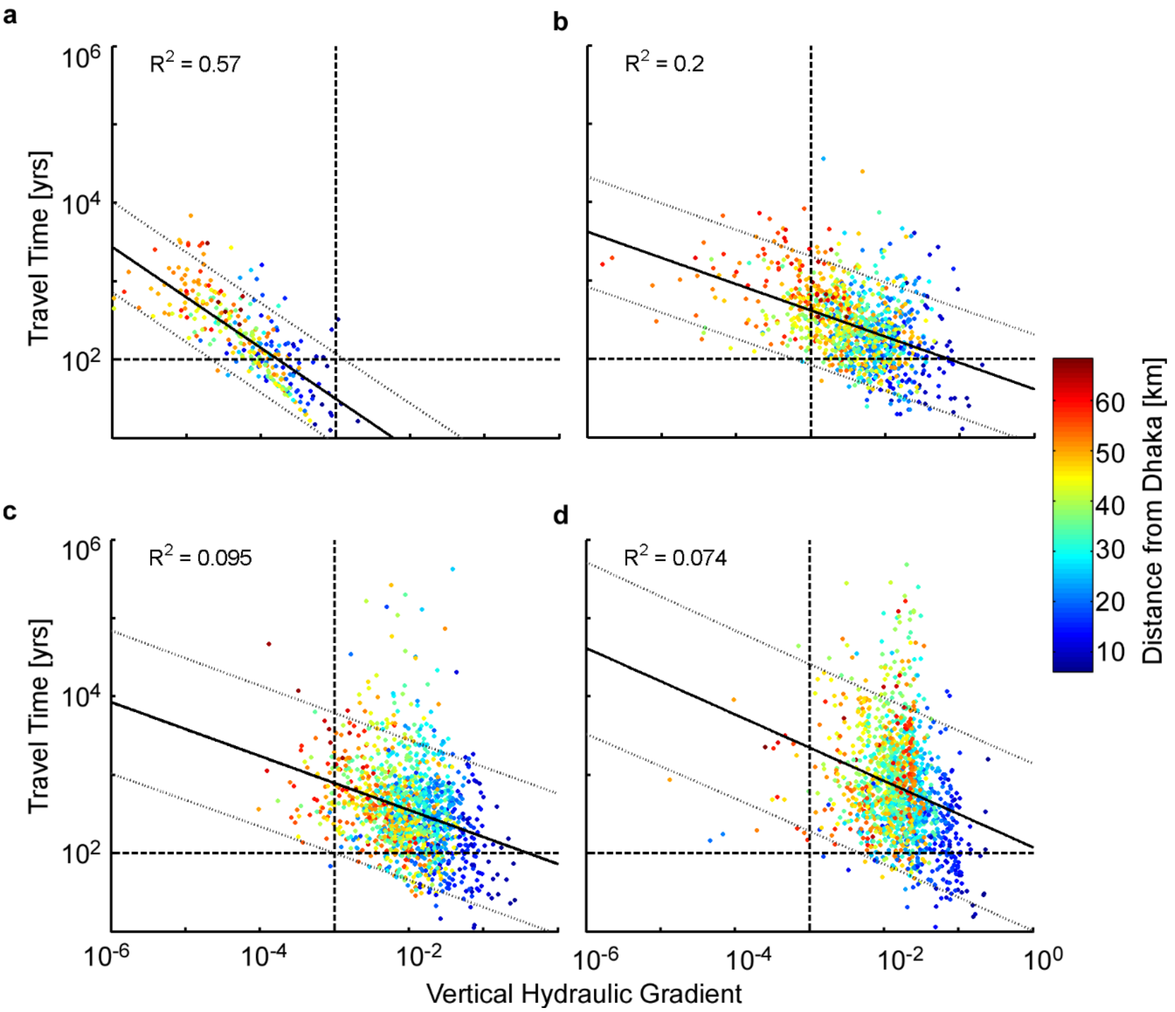

Supplementary Figure 11| Effect of hydraulic gradient and lithology on travel time. Travel time as a function of vertical hydraulic gradient for different ranges of fine sediment thickness (**a**) < 5m, (**b**) ≥5 to <25 m, (**c**) ≥25 to < 50m, and (**d**) ≥ 50 m. The dots are colored according to their distance from the center of Dhaka. The black line is the linear regression and grey lines encompass the 95% confidence interval. The dashed horizontal lines show a travel time of 100 years, while the dashed vertical lines show a vertical hydraulic gradient of 0.001.

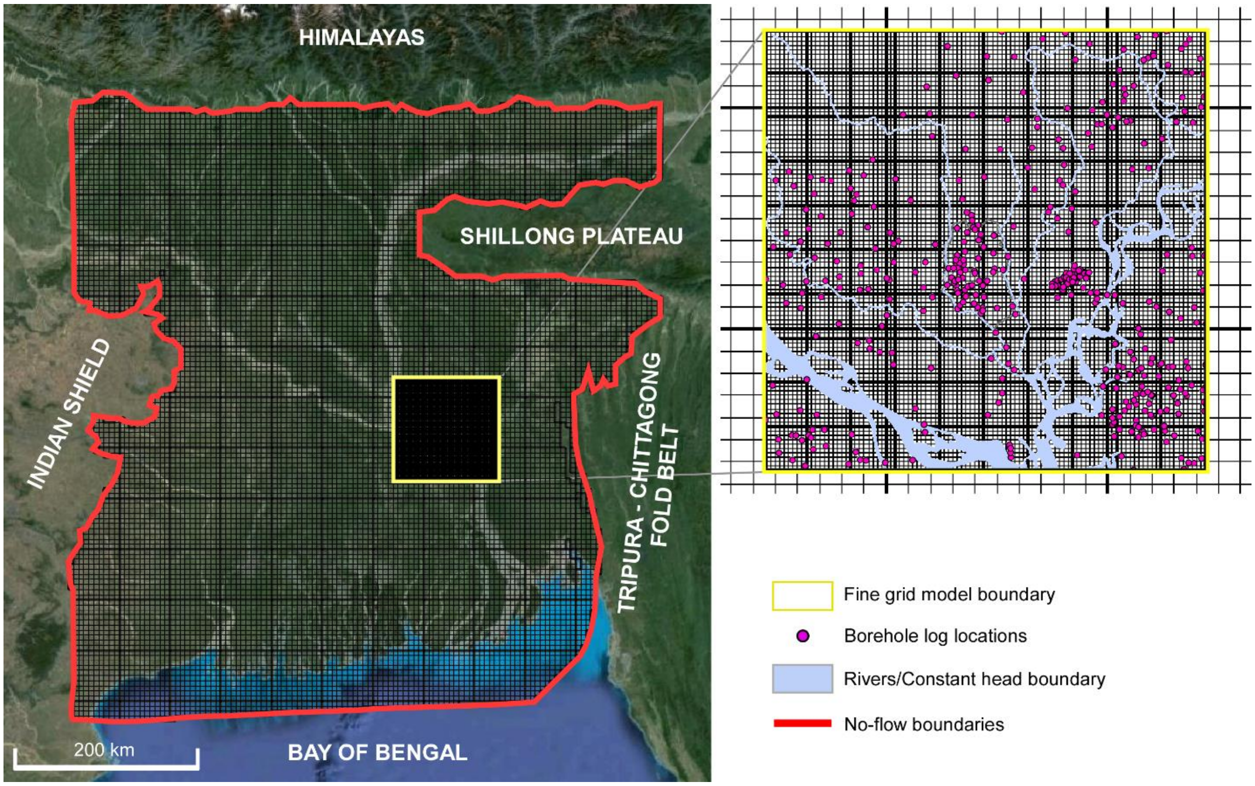

Supplementary Figure 12| Model setup and driller log locations. The Google Earth image on the left shows the coarse model grid and boundary conditions, the map on the right shows the fine-grid model setup and the locations of the driller logs used in the geostatistical simulation of aquifer heterogeneity. A constant head boundary was assigned along the top of the coarse-grid model. Constant head boundaries along the rivers, a recharge boundary, and a drain boundary were assigned along the top of the fine-grid model. The heads along the shared boundaries between the coarse and fine-grid model were calculated iteratively using the MODFLOW-LGR2 package.

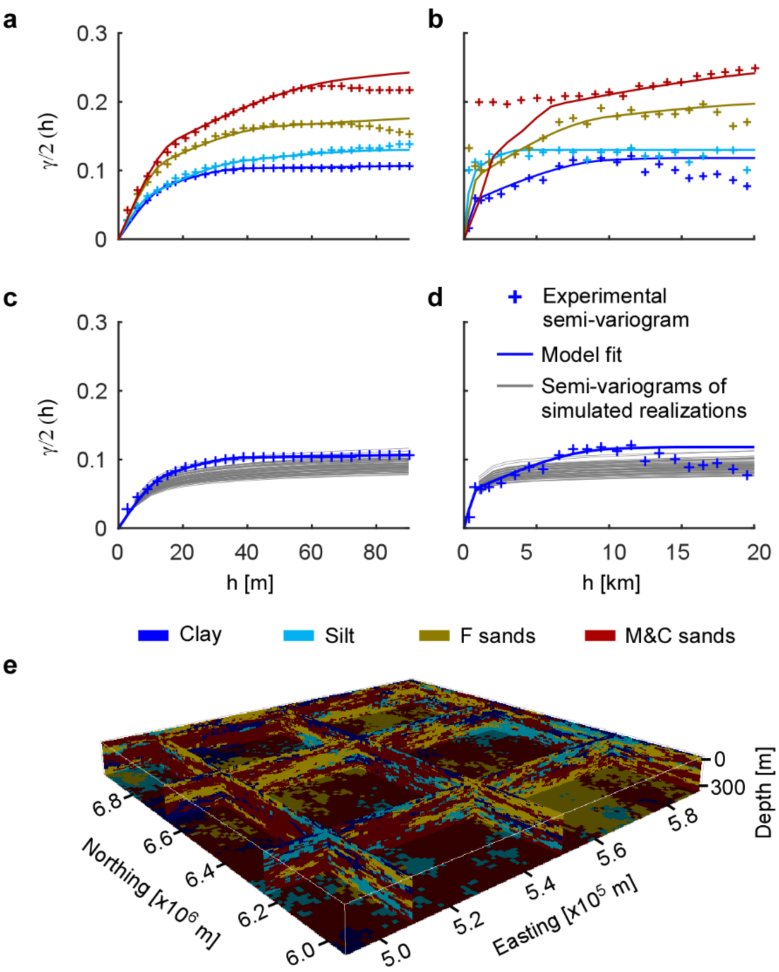

Supplementary Figure 13| Geostatistical simulation results. Experimental semi-variograms and model fit in the (**a**) vertical and (**b**) horizontal directions. Comparison of the input variogram model with the variogram of the simulated realizations for Clays in (**c**) vertical and (**d**) horizontal directions. (**e**) Example of a simulated field of heterogeneity (Realization 1), color legend applies for all panels.

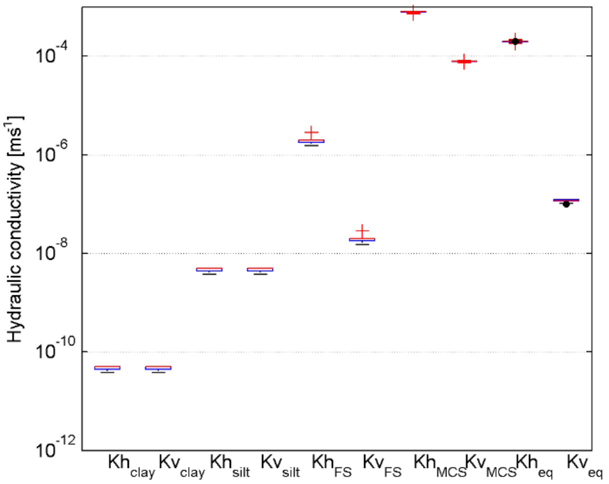

Supplementary Figure 14| Ranges in facies hydraulic conductivity and Darcy equivalent hydraulic conductivity values for 60 heterogeneous realizations. Darcy equivalent horizontal and vertical hydraulic conductivity values of the equivalent homogeneous system are shown as black dots. Box plots show median (red line), range (whiskers), and outliers (red +).

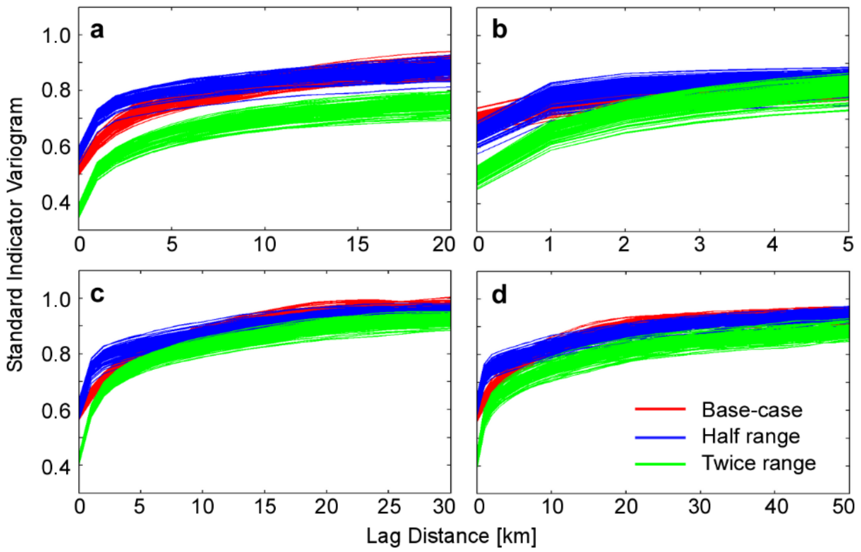

Supplementary Figure 15| Comparison of different lithofacies variograms of the simulated realizations in three different cases considered in this study. Realization variograms of Clay (**a**), silts (**b**), fine sands (**c**), and medium and coarse sands **(d**).

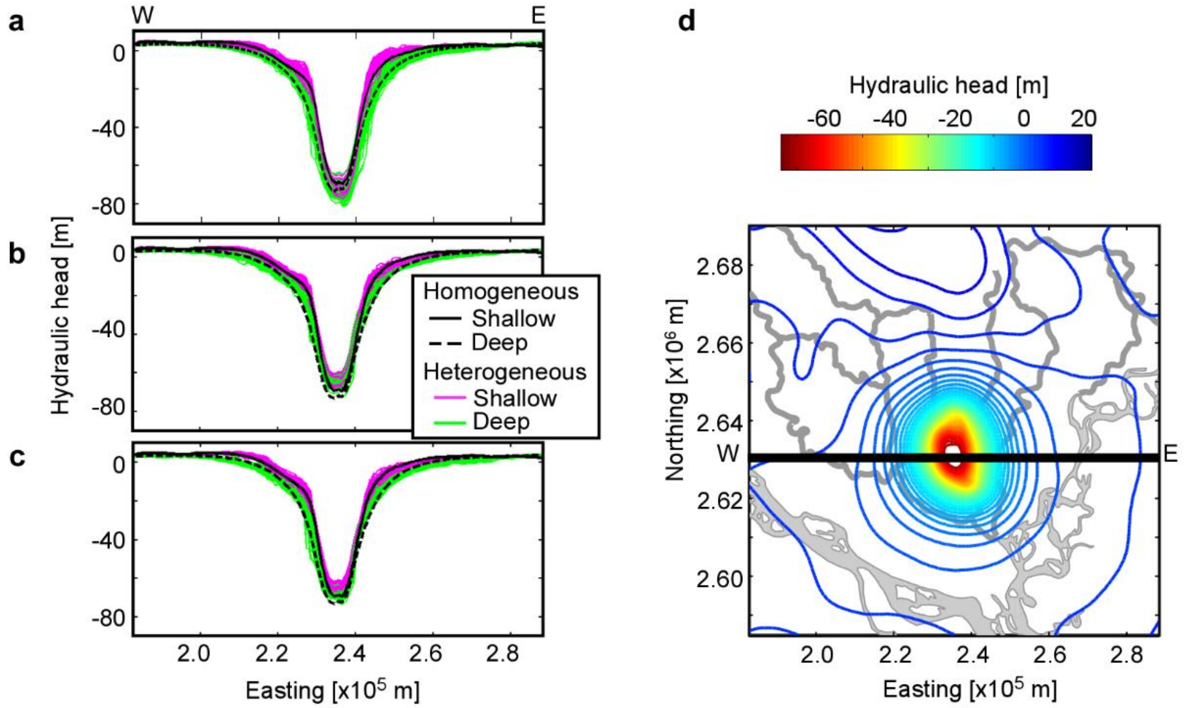

Supplementary Figure 16| Comparisons of simulated hydraulic heads for the three horizontal variogram ranges considered. Heads at a depth of 50 m (shallow) and 150 m (deep) for the base case (**a**), half-range case (**b**), and twice-range case (**c**). The transect location is shown in (**d**). Contour lines in (**d**) show the simulated hydraulic head at a depth of 150 m for the homogeneous model.

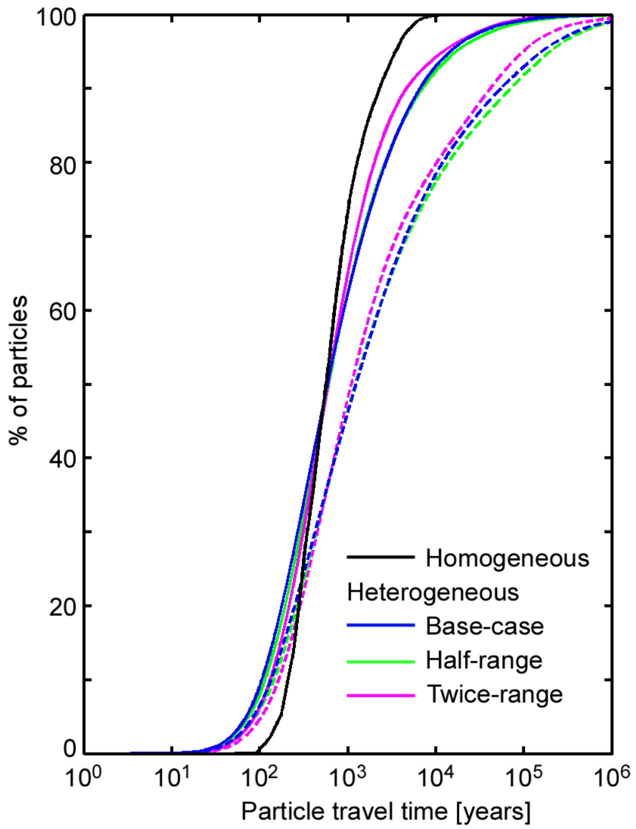

Supplementary Figure 17| Comparison of breakthrough curves for the three for the three horizontal variogram ranges considered. Travel time to 150 m depth from 50 m depth based on backward particle tracking. For the heterogeneous cases, the solid lines and the dashed lines are cumulative distribution functions of the ensemble of all sixty realizations for sand only particles and all particles, respectively.

Supplementary Table 1| Hydraulic conductivity values used for different lithofacies.

	Medium and coarse sands		Very fine and fine sands		Silts		Clays		Equivalent	
	K_h	K_h/K_v	K_h	K_h/K_v	K_h	K_h/K_v	K_h	K_h/K_v	K_h	K_h/K_v
Half Range	7.0×10^{-4} (±12%)	10	1.6×10^{-6} (±22%)	100	3.9×10^{-9} (±22%)	1	3.9×10^{-11} (±22%)	1	2.0×10^{-4} (±5%)	1.0×10^{-7} (±14%)
Base Case	7.7×10^{-4} (±5%)	10	2.2×10^{-6} (±30%)	100	4.4×10^{-9} (±13%)	1	4.4×10^{-11} (±13%)	1	2.0×10^{-4} (±8%)	1.1×10^{-7} (±9%)
Twice Range	5.7×10^{-4} (±13%)	10	1.6×10^{-6} (±20%)	100	4.1×10^{-9} (±20%)	1	4.1×10^{-11} (±20%)	1	2.1×10^{-4} (±4%)	1.0×10^{-7} (±9%)

Supplementary Table 2| Summary particle travel time statistics. The statistics are based on only those particles that were originally placed in sands and ended up inside the fine-grid model. Travel times are in years.

	Homogeneous	Ensembles mean Heterogeneous realizations		
		Half Range	Base Case	Twice Range
Minimum	89	8	7	11
Mean (Fastest 10%)	131	72	67	84
1st Quartile	291	238	220	260
Median	496	605	589	593
Mean	747	7426	6736	10984
3rd Quartile	846	1922	1959	1538
Maximum	1.2×10^4	1.2×10^7	1.5×10^7	5.1×10^7
% particles with <100 year travel time	0	6	9	8
